# Atypical negative pressure pulmonary edema after extracorporeal membrane oxygenation therapy for COVID‐19

**DOI:** 10.1002/rcr2.1071

**Published:** 2022-12-08

**Authors:** Yu Suzuki, Takaaki Ogoshi, Yusuke Taura, Shiori Oda, Daiji Uchiyama, Hiroyuki Ueda, Kazuhiro Yatera

**Affiliations:** ^1^ Department of Respiratory Medicine Kokura Memorial Hospital Kitakyushu‐shi Japan; ^2^ Department of Otorhinolaryngology Kokura Memorial Hospital Kitakyushu‐shi Japan; ^3^ Department of Diagnostic Radiology Kokura Memorial Hospital Kitakyushu‐shi Japan; ^4^ Department of Respiratory Medicine University of Occupational and Environmental Health Kitakyushu‐shi Japan

**Keywords:** ARDS, COVID‐19, negative pressure pulmonary edema

## Abstract

NPPE imaging findings were reported to show a preferential central and nondependent distribution. However, in our case, NPPE showed a peripheral accent pattern, resembling the ARDS pattern of COVID‐19 pneumonia 4 months ago. Capillary damage from COVID‐19 might still exist.

## Graphical abstract

Atypical negative pressure pulmonary edema showing a peripheral accent pattern resembling ARDS has emerged after COVID‐19 pneumonia four months ago. Capillary damage from COVID‐19 might still exist.

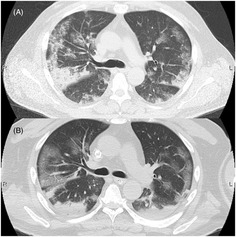

## CLINICAL IMAGE

**FIGURE 1:**
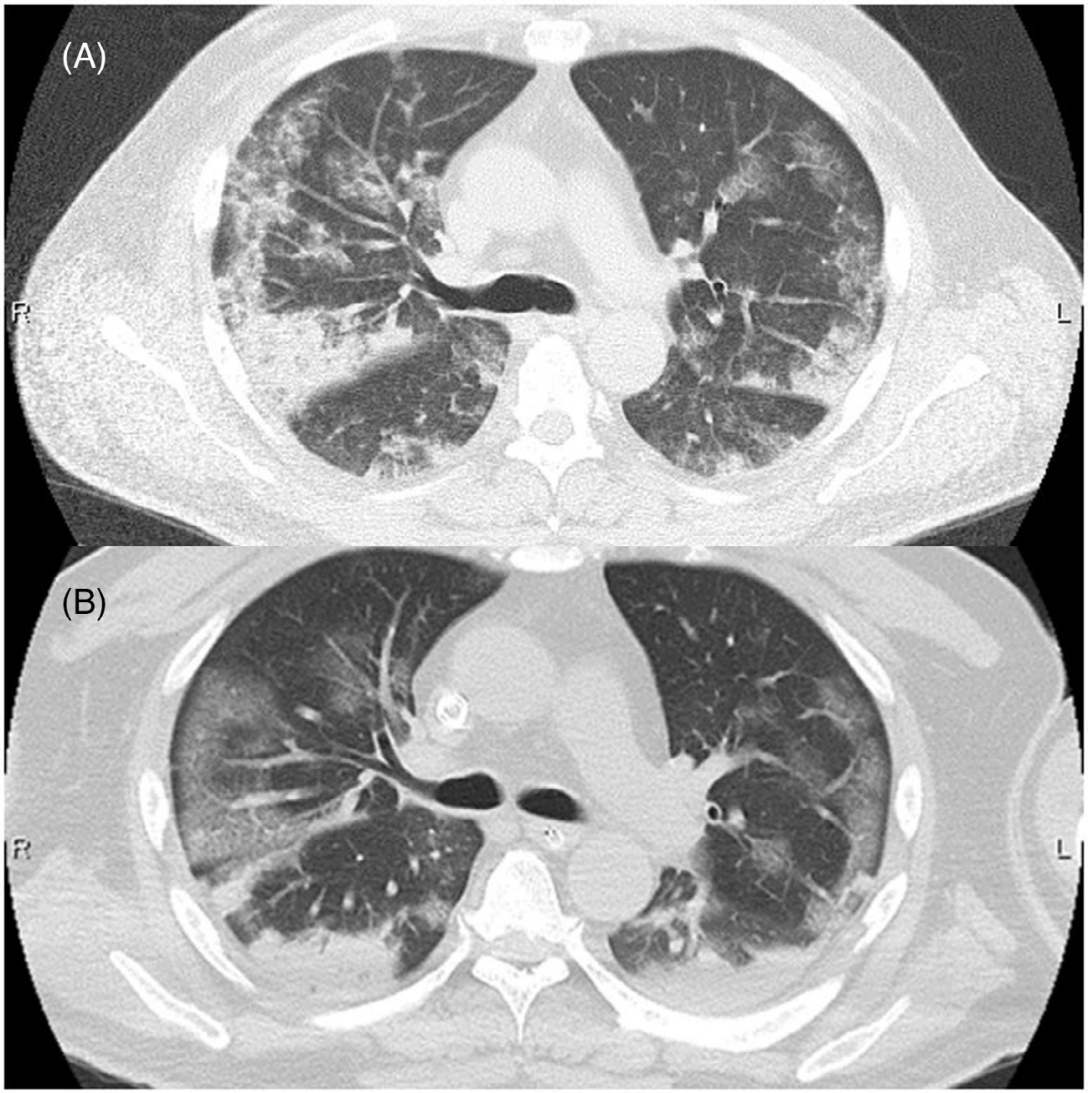
(A) An axial thoracic CT image obtained soon after recovery showing bilateral patchy ground‐glass opacities and accented non‐segmental shadows in the subpleural region. (B) An axial thoracic CT image obtained 4 months previously, immediately after intubation for ECMO therapy, showing typical imaging findings of SARS‐CoV‐2 pneumonitis with ARDS

**FIGURE 2:**
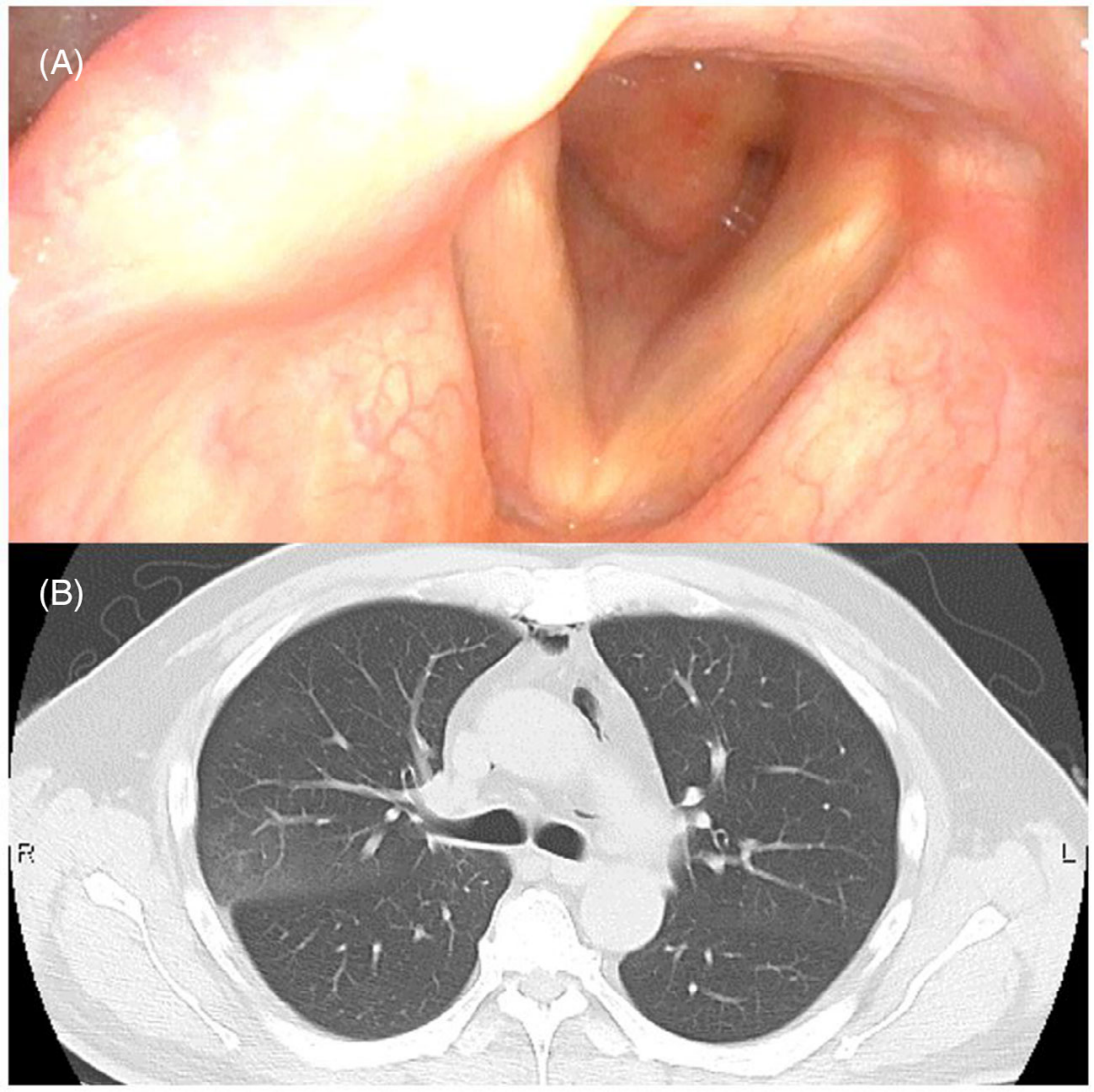
(A) Pharyngeal fibroscopy demonstrates subglottic granuloma spreading to the vocal cords. (B) An axial thoracic CT image obtained 4 days after emergent tracheostomy showing the disappearance of pulmonary edema

A 53‐year‐old man presented to our emergency room 4 months after undergoing extracorporeal membrane oxygenation (ECMO) for 12 days due to SARS‐CoV‐2 (COVID‐19) infection. He lost consciousness following rapid‐onset hyperventilation with stridor and a decrease in oxygen saturation. While breathing under resting conditions, he recovered spontaneously without intubation, and initial computed tomography (CT) showed bilaterally‐scattered patchy ground‐glass opacities and consolidations with an accented subpleural non‐segmental distribution (Figure 1A), similar to the previous COVID‐19 pneumonitis and acute respiratory distress syndrome pattern (Figure 1B). COVID‐19 polymerase chain reaction was negative. Cervical CT and pharyngeal fibroscopy revealed subglottic granuloma due to previous intubation (Figure 2A). Pulmonary shadows spontaneously resolved 4 days later, without steroids, antibiotics, or diuretics (Figure 2B). Given this clinical course, the pulmonary shadows were attributed to negative pressure pulmonary edema (NPPE). Acute respiratory failure after intense inspiratory effort against an obstructed airway has been well‐described as NPPE, and some authors have discussed the differential diagnoses of NPPE and COVID‐19 pneumonitis.^1^, ^2^ Typical NPPE imaging findings reportedly involve preferential central and nondependent distribution without exception. This atypical type of NPPE would have been a very rare case before the COVID‐19 pandemic. There may have been persistent peripheral capillary damage from COVID‐19.

## AUTHOR CONTRIBUTIONS

Yu Suzuki was responsible for the conceptualization and drafting of the manuscript. Takaaki Ogoshi and Yusuke Taura revised it critically for important intellectual content. Shiori Oda performed surgical treatment, and Daiji Uchiyama and Hiroyuki Ueda analysed and interpreted the CT findings. All authors, including Kazuhiro Yatera, read and approved the final manuscript.

## CONFLICT OF INTEREST

None declared.

## ETHICS STATEMENT

The authors declare that appropriate written informed consent was obtained for the publication of this manuscript and accompanying images.

## Data Availability

The data that support the findings of this study are available from the corresponding author upon reasonable request.

## References

[1] Holzgreve A , Fabritius MP , Conter P . CT findings in negative pressure pulmonary edema. Diagnostics (Basal). 2020;10(10):749. 10.3390/diagnostics10100749 PMC7599785 32992752

[2] Karaman I , Ozkaya S . Differential diagnosis of negative pressure pulmonary edema during COVID‐19 pandemic. J Craniofac Surg. 2021;32(5):e421–3. 33201071 10.1097/SCS.0000000000007226 PMC8237840

